# Immunoglobulin G glycosylation and its alterations in aging-related diseases: IgG glycosylation with aging-related diseases

**DOI:** 10.3724/abbs.2024137

**Published:** 2024-08-08

**Authors:** Yongqi Wu, Zhida Zhang, Lin Chen, Shisheng Sun

**Affiliations:** Laboratory for Disease Glycoproteomics College of Life Sciences Northwest University Xi’an 710069 China

**Keywords:** IgG, glycosylation, inflammation, aging, aging-related diseases

## Abstract

Immunoglobulin G (IgG) is an important serum glycoprotein and a major component of antibodies. Glycans on IgG affect the binding of IgG to the Fc receptor or complement C1q, which in turn affects the biological activity and biological function of IgG. Altered glycosylation patterns on IgG emerge as important biomarkers in the aging process and age-related diseases. Key aging-related alterations observed in IgG glycosylation include reductions in galactosylation and sialylation, alongside increases in agalactosylation, and bisecting GlcNAc. Understanding the role of IgG glycosylation in aging-related diseases offers insights into disease mechanisms and provides opportunities for the development of diagnostic and therapeutic strategies. This review summarizes five aspects of IgG: an overview of IgG, IgG glycosylation, IgG glycosylation with inflammation mediation, IgG glycan changes with normal aging, as well as the relevance of IgG glycan changes to aging-related diseases. This review provides a reference for further investigation of the regulatory mechanisms of IgG glycosylation in aging-related diseases, as well as for evaluating the potential of IgG glycosylation changes as markers of aging and aging-related diseases.

## Introduction

Aging is a multifaceted biological process involving a gradual overall decline in physiological function and homeostasis over time, making individuals increasingly susceptible to a spectrum of chronic and degenerative conditions [1]. This progressive decline encompasses various cellular, molecular, and systemic changes, including genomic instability, telomere shortening, epigenetic alterations, mitochondrial dysfunction, and proteostasis and inflammatory pathway dysregulation [2]. Aging is jointly regulated by a variety of genetic and epigenetic factors, including the abnormal expressions of aging-related genes, increased DNA methylation levels, altered histone modifications, and disrupted protein translation homeostasis [3]. This regulation exhibits significant variability, heterogeneity, and plasticity, reflecting the complex interplay between genetic predispositions and epigenetic influences [3]. Age-related diseases, such as Alzheimer’s disease, cardiovascular disease, metabolic disorders, and chronic inflammation, represent significant burdens on global health and pose formidable challenges to healthcare systems worldwide [4]. These diseases often share common risk factors and pathological mechanisms with aging, suggesting intricate connections between the aging process and disease pathogenesis [ 5, 6].

Protein glycosylation refers to the enzymatic process of attaching carbohydrate molecules via covalent bonds to specific functional groups on proteins [7]. The “paracentral dogma” hypothesis, which proposes glycosylation as a third fundamental life code after DNA/RNA and proteins [8], underscores the crucial role of sugar codes in co- and post-translational modifications (PTMs). Glycosylation enhances the classical central dogma by providing an additional layer of regulation and information that is crucial for protein folding, stability, cell‒cell communication, and signaling. Protein glycosylation, which is influenced by both genetic and epigenetic factors, plays a pivotal role in numerous cellular signaling and communication events [9]. Glycomedicine refers to glycomics- and glycoproteomics-based biomarkers and therapeutic target discovery; therefore, it has great potential to provide a new dimension of medical science toward better disease diagnosis and drug discovery [10].

Dysregulation of *N*-glycosylation has been implicated in the aging process and age-related diseases [ 11‒ 13]. In particular, increasing evidence has shown that significant glycan structure alterations in immunoglobulins (Igs) are closely associated with human aging and various aging-related diseases [13]. Immunoglobulin G (IgG) *N*-glycosylation is thought to be the link between the genetic code and the cellular environment. The environment can strongly influence the inflammatory properties (pro- or anti-inflammatory) of IgGs by regulating their glycosylation. Given the high abundance and important immune functions of IgG in the human body, elucidating the changes in IgG glycosylation associated with aging and aging-related diseases is crucial for obtaining a better understanding of human aging and its associated diseases.

**Figure 1:**
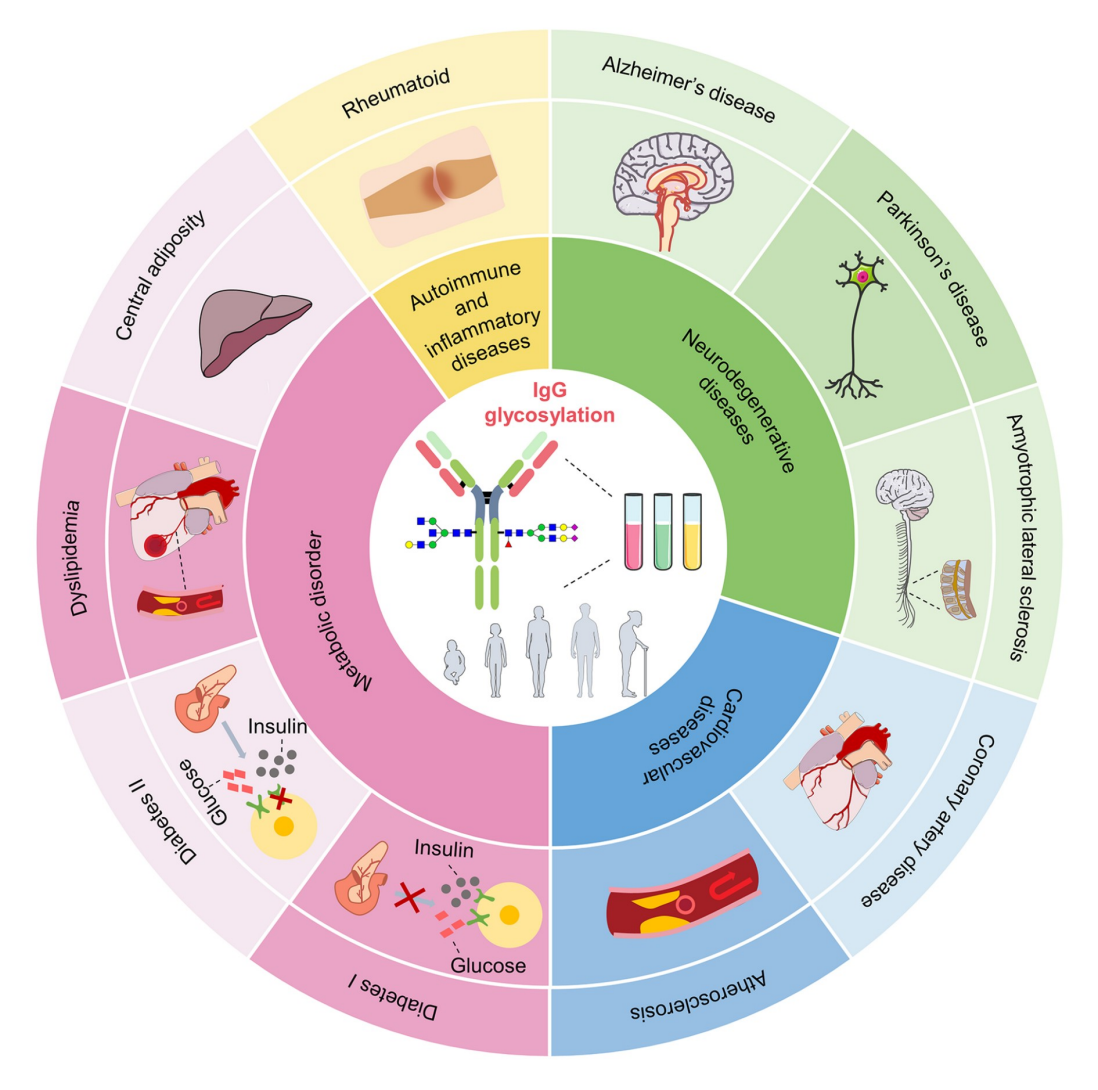
Figure 1 Overview of IgG glycosylation alterations associated with aging and aging-related diseases

Several excellent reviews have summarized the role of IgG glycosylation in the immune system [14], inflammation [15], cancer [16], and infectious diseases [17]. A review also discussed the potential roles of IgG glycosylation as a marker for biological aging [18]. In this review, we further summarize recent research advances on IgG glycosylation alterations associated with aging and aging-related diseases ( Figure 1), the effects of glycosylation on IgG functions, and the pathogenesis of age-related diseases. In addition, the potential of IgG glycosylation changes as biomarkers for the diagnosis and treatment of aging and age-related diseases is discussed. This review will help readers understand the relationship between IgG glycosylation changes and aging-related diseases and provide important insights and directions for future research in the field of aging glycobiology.

## Overview of IgG

Immunoglobulins play an important role in the adaptive immune response. They consist of two identical light and heavy chains linked by disulfide bonds to form a nearly “Y”-shaped structure. Each heavy chain contains 3‒4 constant regions (CH) and one variable region (VH), while each light chain contains one constant region (CL) and one variable region (VL). Each variable region contains three highly variable ring-structured complementary decision regions (CDRs), and six CDRs in each arm of the antibody’s Y-shaped structure work in concert to form an antigen-binding site, so the Y-shaped arm region of the antibody is known as the antigen-binding region (Fab), which operates like a signal receiver. The fragment crystallizable region (Fc), which consists of two or three constant structural domains per heavy chain [19], transmits signals downstream to various effector cells of the immune system. The Fab region recognizes and binds to specific antigens, while the Fc region interacts with different Fcγ receptors expressed on the surface of immune effector cells to activate downstream pathways [ 20, 21].

**Figure 2:**
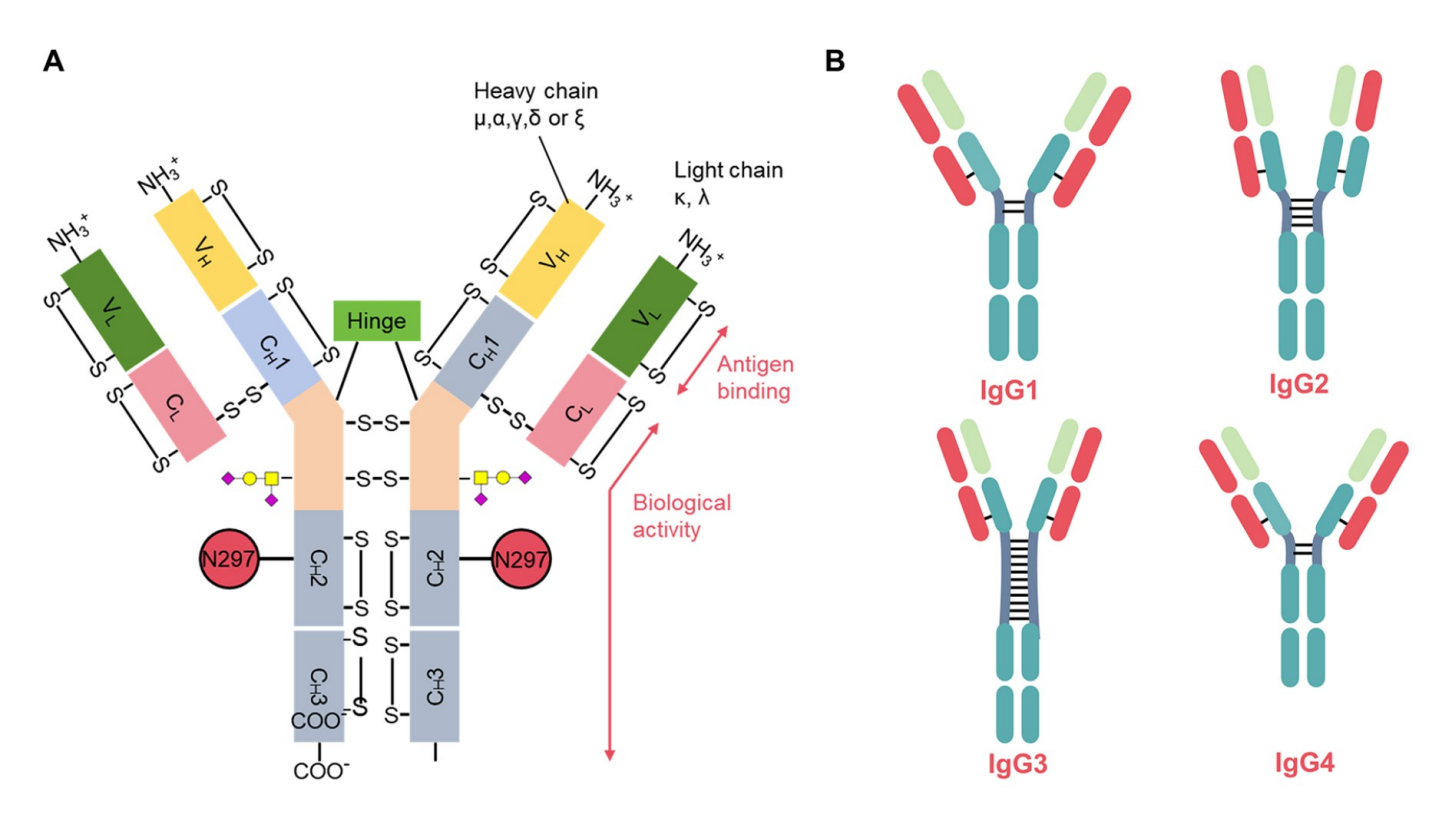
Figure 2 IgG basic structure and subclasses (A) Schematic representation of IgG. (B) Four subclasses of IgG, namely, IgG1, IgG2, IgG3, and IgG4.

There are five isotypes of immunoglobulins (IgM, IgD, IgG, IgA, and IgE) in humans, which are distinguished by the C-terminal region of the heavy chain [22] ( Figure 2A). IgG, which is normally secreted by B cells and plasma cells and is present as a monomer, is the predominant immunoglobulin (~75%) and the most abundant glycoprotein (~10 mg/mL) in human serum [ 23, 24]. There are four subclasses or isotypes of IgG antibodies in human (IgG1–4) and mouse (IgG1, IgG2a, IgG2b, and IgG3) [25]. IgG distribution abundance in human serum is as follows: IgG1 (60%), IgG2 (25%), IgG3 (10%), and IgG4 (5%). The number of disulfide bonds in the hinge region varies among IgG isoforms, with IgG1 and IgG4 containing two interchain disulfide bonds in the hinge region, IgG2 having four disulfide bonds, and IgG3 having 11 disulfide bonds [26] ( Figure 2B). Crystallographic data for the whole IgG molecule are still limited because the flexible nature of the hinge region prevents crystallization, unlike the cumulative crystal structure of Fab and Fc fragments in the complex. To date, only four crystal structures have provided entire views of the IgG structure: PDB codes 1igt, 1igy, 1hzh, and 5dk3 for mouse IgG1, mouse IgG2a, human IgG1, and humanized IgG4 structures, respectively [ 27‒ 30]. The biological functions of these subtypes are also different. IgG1 is produced primarily through the induction of responses to soluble protein antigens and membrane proteins, and a lack of IgG1 leads to a decrease in overall IgG levels [ 31, 32]. IgG2 is the only antibody that can respond to bacterial capsule polysaccharide antigens. Increased susceptibility to certain bacterial infections is associated with IgG2 deficiency, which suggests that IgG2 plays a role in defending against these pathogens [33]. IgG3 antibodies are particularly effective in the induction of effector functions. As a potent pro-inflammatory antibody, its shorter half-life may function to limit the potential for excessive inflammatory responses [34]. IgG4 antibodies are usually formed after repeated or long-term exposure to antigens in non-infected environments and may become major subclasses [35]. The structure, function, antigen binding, immune complex formation, complement activation, effector cell triggering and half-life of the different IgG subtypes have been described in detail in previous reviews and will not be repeated and discussed further here [25].

## IgG Glycosylation

**Figure 3:**
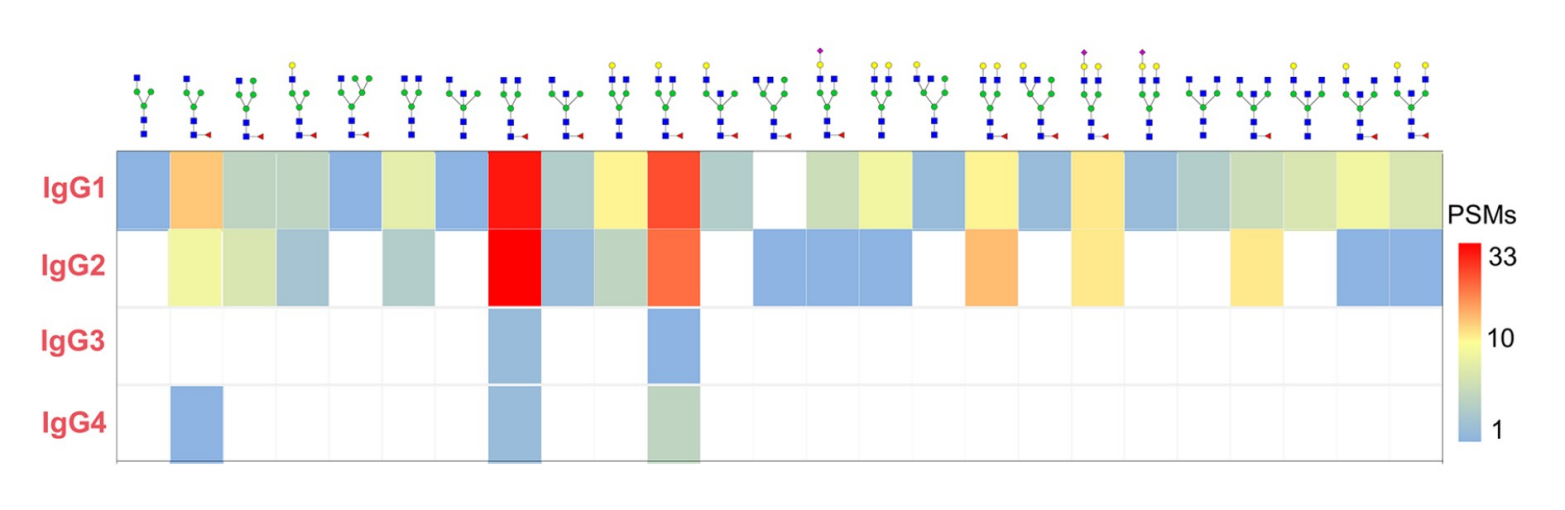
Figure 3 *N*-Glycan structures of human IgG [40]

Glycosylation, one of the most common post-translational modi-fications in mammalian cells, impacts many biological processes, such as cell adhesion, proliferation and differentiation. There are two main types of glycosylation of proteins in mammals: *N*-glycosylation and *O*-glycosylation [36]. Notably, the structure and effector functions of IgG are regulated by *N*-glycosylation, including galactosylation, sialylation, core-fucosylation, as well as bisecting GlcNAcylation, which will be further described in details [ 21, 37]. *N*-glycosylation involves the attachment of glycans to the asparagine (Asn) side chain nitrogen atom, specified by the consensus sequence Asn–X–Ser/Thr (X=amino acid except proline) [23]. N-Linked glycans can be found at the Fab and Fc fragments of all IgG subclasses [ 38, 39]. IgG has a conserved *N*-glycosylation site at Asn297 in the CH2 domain of the Fc region. Our team previously conducted a comprehensive characterization of the *N*-glycosylations of the four IgG isoforms using self-developed StrucGP software, in which 25 *N*-glycan structures were identified in IgG1, whereas fewer *N*-glycan structures were identified in IgG2-4 than in IgG1 [40]. Most of the identified site-specific glycans (84.6%) were core fucosylated, which is consistent with previous studies [ 41, 42] ( Figure 3). Depending on their presence and composition, antigen binding and effector functions such as phagocytosis, complement activation and inflammatory processes are induced with varying effectiveness [ 20, 43]. In addition, N- and O-linked glycans were found in the Fc fusion protein atacicept [44]. *O*-glycosylation of the amino acid serine (Ser) or threonine (Thr) is rare in IgG. *O*-glycosylation has been reported for various immunoglobulins. *O*-glycans are found in the hinges of human IgA1, IgD and mouse IgG2b [ 45‒ 47]. *O*-glycosylation in the hinge of murine IgG2b was found to protect against proteolytic digestion. A recent study reported that partial *O*-glycans were detected on threonine residues in the hinge zone of IgG3 [48]. There is a paucity of information regarding IgG *O*-glycosylation. In this paper, we will primarily focus on the *N*-glycosylation of immunoglobulin G.

### Biosynthesis of *N*-glycans on IgG

**Figure 4:**
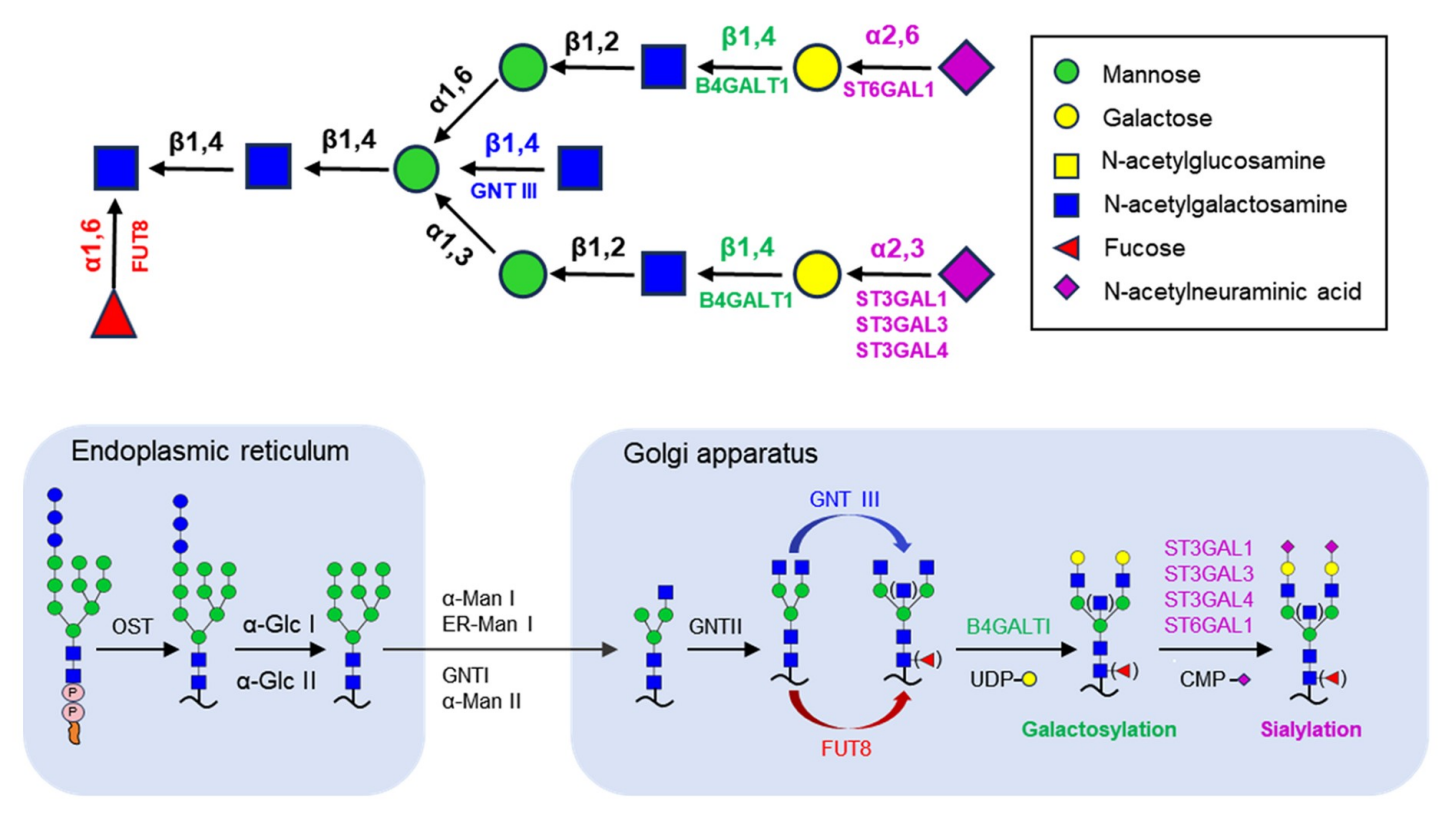
Figure 4 Brief schematic diagram of the IgG *N*-glycan synthesis pathway

Protein glycosylation is a complex, multistep process that involves the use of approximately 200 glycosyltransferases that determine which proteins are glycoproteins. IgG *N*-glycan attachment and maturation occur in the endoplasmic reticulum and Golgi apparatus with the help of glycotransferases and glycosidases [49]. N-linked glycosylation in most eukaryotes follows a similar initial processing pathway within the endoplasmic reticulum. In this paper, we will provide a brief description of the biosynthesis pathway of *N*-glycans on IgG. This pathway begins with the generation of a lipid linked oligosaccharide (LLO) by multiple asparagine-linked *N*-glycosylation processing enzymes (ALGs). Lipoglycan precursors (Glc3Man9GlcNAc2 oligosaccharides) are transferred to the glycosylation site Asn297 of the IgG heavy chain by multisubunit oligosaccharyltransferase (OST) of the endoplasmic reticulum. After moving to the cis-Golgi apparatus, three mannose residues are trimmed by α-Man I, and one GlcNAc from UDP-GlcNAc is added to the terminal mannose residue at the α1-3 branch of the oligosaccharide by GnT-II. In addition, FUT8 transfers the fucose moiety from GDP-β-L-fucose to the innermost GlcNAc residue in the *N*-glycan, and a “bisecting” GlcNAc can be added to the innermost Man residue by one of many GlcNAc transferases (GNTIII) that play a role in generating branches. These antennae are further extended by the addition of galactose (Gal) residues via β-1,4 linkages. These branches can be further modified in several ways, including GlcNAc-Gal extensions (LacNAc) or the addition of a second Gal residue in some mammals via an α-1,3 linkage, which can elicit an immune response in humans. The β-1,4-linked Gal residues are then often capped with sialic acid via α-2,3 or α-2,6 linkages [ 7, 50] ( Figure 4).

### Galactosylation

Galactosylation occurs in the Golgi apparatus and is mediated by galactosyltransferase, which works by catalyzing the linkage of galactose in uridine diphosphate galactose (UDP-Gal) to N-acetylglucosamine. The enzyme β1,4-galactosyltransferase, also called β-N-acetylglucosaminyl-glycopeptide β1,4-galactosyltransferase, is a trans-Golgi-resident enzyme [51]. The level of galactosylation of IgG changes slowly and progressively with aging. The abundance of agalactosylated IgG *N*-glycans increased from 20% at 25 years of age to 40% by 70 years of age, peaking in >90-year-old subjects [ 52‒ 55]. IgG galactosylation appears to be, at least in part, driven by estrogen concentrations. This is evidenced by increased levels during pregnancy and decreased levels after menopause. In addition, an endocrine manipulation study confirmed that estrogen is an *in vivo* regulator of IgG galactosylation in both females and males, suggesting a gender-dependent mechanism of immune response regulation [ 54, 56, 57].

### Sialylation

Sialylation involves the addition of terminal sialic acid to cell membrane glycoproteins and is mediated by sialyltransferases (ST3GAL/ST6GAL α2,3/α2,6-sialyltransferase), which are involved in embryonic development, neurodevelopment, reprogramming, tumorigenesis and immune response processes. Sialylated Fc *N*-glycans are mediators of anti-inflammatory processes; however, the underlying mechanisms are still under discussion [58]. Studies have shown significantly higher levels of sialylation (especially disialylation) in Fabs than in fragment crystallizable (Fc) glycans. Studies in pediatric populations analyzing mainly Fc glycans have revealed a decrease in the abundance of sialic acid glycans up to 10 years of age, after which the trend may reverse, accompanied by dynamic changes in the abundance of galactosylated glycans. One study found no age-related differences in total IgG sialylation levels, suggesting that age-related changes are driven by changes in Fc chain glycan abundance [ 39, 59].

### Core fucosylation

Core fucosylation, which is catalyzed by alpha-(1,6)-fucosyltransferase 8 (FUT8), is particularly common in mammals. FUT8 transfers fucose from GDP-β-L fucose to the GlcNAc residue in the innermost layer of the *N*-glycan [60]. In contrast to most other plasma proteins that are not core fucose-modified, more than 90% of serum IgGs contain the core fucose modification of the first N-acetylglucosamine (GlcNAc) in the core structure of the IgG glycan [ 61, 62]. A slight decrease in core fucosylation on IgGs has been reported in adolescents compared with children [ 59, 63], implying that alterations in core fucosylation might be associated with aging.

### Bisection

Bisection refers to the attachment of a GlcNAc residue to the core beta-mannose residue of an *N*-glycan with β1,4-linkage and is catalyzed by β1,4-N-acetylglucosaminyltransferase III (also known as GnT-III, GlcNAcT-III or MGAT3). Bisected GlcNAc plays a regulatory role in the biosynthesis of complex and hybrid types of oligosaccharides [ 64‒ 66]. Even though the bisecting glycans account for only a small proportion of IgG glycans (approximately 10%–15%) [39] and no significant changes have been observed in most age-related studies, a slightly elevated level of bisecting linkages in adult males but not in females was reported in a study published in 2011 [67].

## Changes in IgG Glycosylation Mediate Body Inflammation

The ability of IgG antibodies to mediate effector functions arises from their capacity to bridge antigen binding through the Fab domain with the recruitment of effector cells through interactions between the Fc domain and the Fcγ receptor (FcγR) family. Within the CH2 domain, IgG possesses recognition regions for the initial complement protein C1q as well as the FcγR family. FcγRs are composed of type I (FcγRI, FcγRIIa, FcγRIIb, FcγRIIIa, and FcγRIIIb) and type II receptors (DC-SIGN and CD23) [68]. The composition of glycans in the Fc domain impacts receptor binding. Due to glycan modifications, these two types of receptors can be distinguished by their ability to interact with Fc domains, i.e., binding to activating FcγRs promotes an inflammatory response, whereas binding to inhibitory FcγRs activates an anti-inflammatory response. Differences in the *N*-glycosylation of IgG subclasses and CH2 structural domains may lead to structural variation in IgG Fc structural domains, which leads to human immune diversity through FcγR-mediated cellular functions.

**Figure 5:**
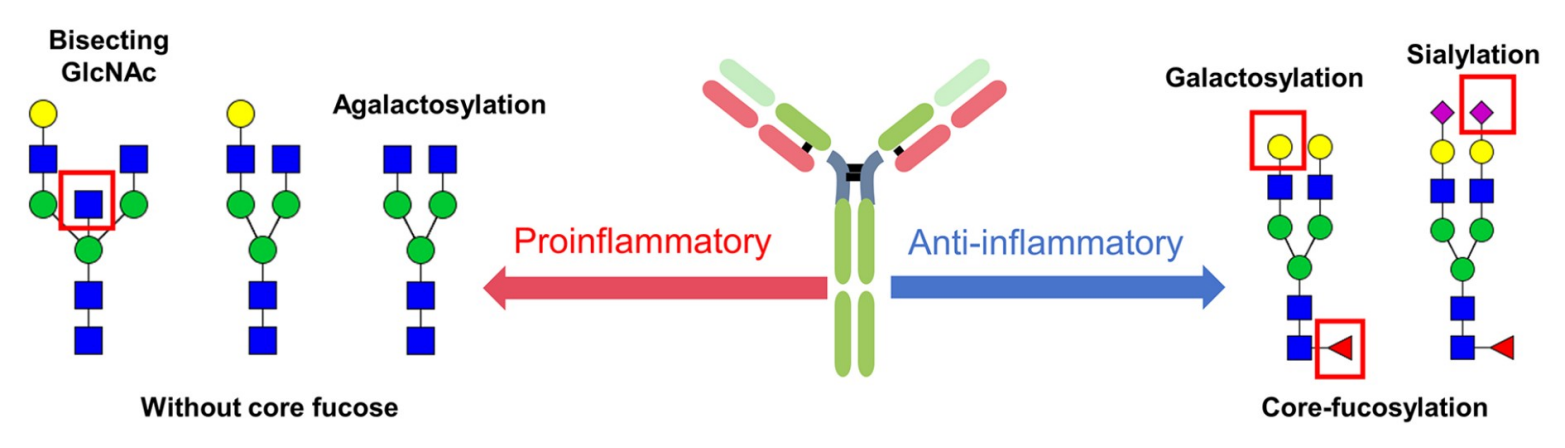
Figure 5 IgG glycosylation and inflammation

It is well known that the addition of each monosaccharide residue to the glycan chain can significantly alter the effect of IgG. A decrease in galactosylation has been observed in many inflammatory diseases, while an increase in galactosylation is usually associated with a decrease in inflammatory activity. This may be due to high galactosylation of IgG by C1q binding to enhance complement activity [ 69, 70]. IgG sialylation has been associated with reduced inflammation and pathological conditions. Specifically, anti-inflammatory activity is driven by antibodies modified with α2,6-sialylation. Fc sialylation reduces binding to Type I FccRs while enabling the engagement of Type II FccRs. In the presence of sialylated Fc glycans, increased Type II FcγR signaling further triggers anti-inflammatory activity [ 14, 71] ( Figure 5). A classic example of this is the administration of high-dose intravenous immunoglobulin (IVIg) during acute inflammatory diseases such as immune thrombocytopenia, Kawasaki disease, chronic inflammatory demyelinating polyneuropathy and Guillain–Barré syndrome. IVIg is pooled IgG from thousands of donors, and its anti-inflammatory activity is mediated by the minor subset of IgGs within the pool that contain sialylated Fcs. Therefore, anti-inflammatory activity can be enhanced by increasing the abundance of sialylated Fc in IgG pools [ 72, 73]. Afucosylated IgG also regulates the inflammatory response via FcγRIIIa and FcγRIIIb. In particular, afucosylated IgG1 has a higher affinity for FcγRIIIa and FcγRIIIb due to an unusual, stabilizing sugar–sugar interaction. The role of afucosylated Fc glycoforms in regulating inflammatory responses has been well demonstrated in dengue infection, where antibodies play a direct role in mediating severe diseases, dengue hemorrhagic fever and dengue shock syndrome [ 14, 74].

IgG glycosylation affects antibody-dependent cellular cytotoxicity (ADCC), complement-dependent cytotoxicity (CDC), antibody-dependent cellular phagocytosis (ADCP), and neutrophil activation, thus demonstrating the critical role of IgG glycans in effector functions [75]. Removal of the core fucose of IgG improves clinical efficacy by enhancing ADCC-mediated killing. Afucosylated IgG has a higher affinity for FcγRIIIA and FcγRIIIB, and ADCC increases 100 times more than fucosylated IgG. This phenomenon is heavily exploited in the production of monoclonal antibodies (mAbs) that are dependent on ADCC processes [76]. The incorporation of bisecting GlcNAc has a similar effect on ADCC, albeit with much weaker potency [ 76, 77]. Increased sialylation of IgG promotes ADCC, leading to the conversion of intravenous immunoglobulins from a pro-inflammatory state to an anti-inflammatory state [69].

## Changes in IgG Glycan with Biological Aging

In recent years, the suggestion that IgG glycans are promising biomarkers for predicting biological age has garnered significant attention in research and clinical fields. Several studies have revealed reasonable correlations between specific IgG glycan structures and an individual’s biological age, suggesting their utility in assessing aging processes. Krištić *et al*. [57] observed a negative association between age and the abundance of monosialylated and disialylated IgG glycans in all examined groups of individuals [ 57, 78]. By utilizing these markers, researchers have begun to predict biological age accurately, offering insights into an individual’s overall aging status and susceptibility to age-related diseases. A new glycomic age index combining one agalactosylated glycan (GP6) and two digalactosylated glycans (GP14 and GP15) has been developed based on the results of the study, although it predicts the actual age with an error of 9.7 years, which is closely related to various biochemical and physiological characteristics reflecting biological aging. Exceptionally, IgG galactosylation is strongly correlated with age, and the development of high-throughput methods for glycan analysis has allowed replication of the results of small-sample studies in cohorts involving thousands of individuals, ultimately confirming an age-related trend in IgG galactosylation, as evidenced primarily by an age-related decrease in digalactosylated and galactosylated IgG glycoforms and a concomitant increase in agalactosylated IgG glycoforms [ 54, 79‒ 81]. This hypothesis is further supported by the observed decrease in galactosylation in some premature aging syndromes [82].

In conclusion, in both men and women, galactosylation and sialylation of IgG decrease with age, with the latter decreasing most dramatically during menopause but showing a transient increase during pregnancy, suggesting hormonal regulation [78]. Studies have shown that fluctuations in estrogen and progesterone levels can lead to variations in the levels of galactosylation and sialylation of IgG. For instance, increased estrogen levels during the follicular phase are associated with increased IgG galactosylation, whereas the luteal phase, characterized by elevated progesterone levels, tends to decrease IgG galactosylation [78]. In addition, IgG glycomic profiles, especially monogalactosylation profiles, are significantly different between and within various ethnic populations [83]. Additionally, research indicates that in addition to alterations in galactosylation and sialylation, other aspects of IgG glycosylation remain relatively stable throughout the human lifespan. Specifically, the levels of bisecting GlcNAc, another type of glycan modification, show minimal changes over time. Similarly, sialylation of the Fc region appears to undergo little variation across different stages of life [ 80, 84]. These findings suggest a nuanced regulatory mechanism governing IgG glycosylation dynamics, where certain modifications are influenced by hormonal fluctuations, while others maintain a steady state despite aging processes. Mouse models are widely used to study the mechanisms of physiological and pathological conditions in humans, which can be used to rule out genetic or environmental influences on IgG glycosylation. An *N*-glycome analysis of IgG in 589 mice with different genetic backgrounds revealed that different strains of mice exhibited certain differences in IgG *N*-glycosylation [85]. The detailed characteristics of IgG *N*-glycosylation in C57BL/6 mice of different ages also provide necessary references for investigating the function of IgG glycosylation in age-related studies [86]. Further investigation into the specific mechanisms underlying these glycosylation patterns using mouse models could provide valuable insights into the interplay between hormonal regulation, aging, and immune function.

## Changes in IgG Glycan Levels in Patients with Aging-Related Diseases

**Figure 6:**
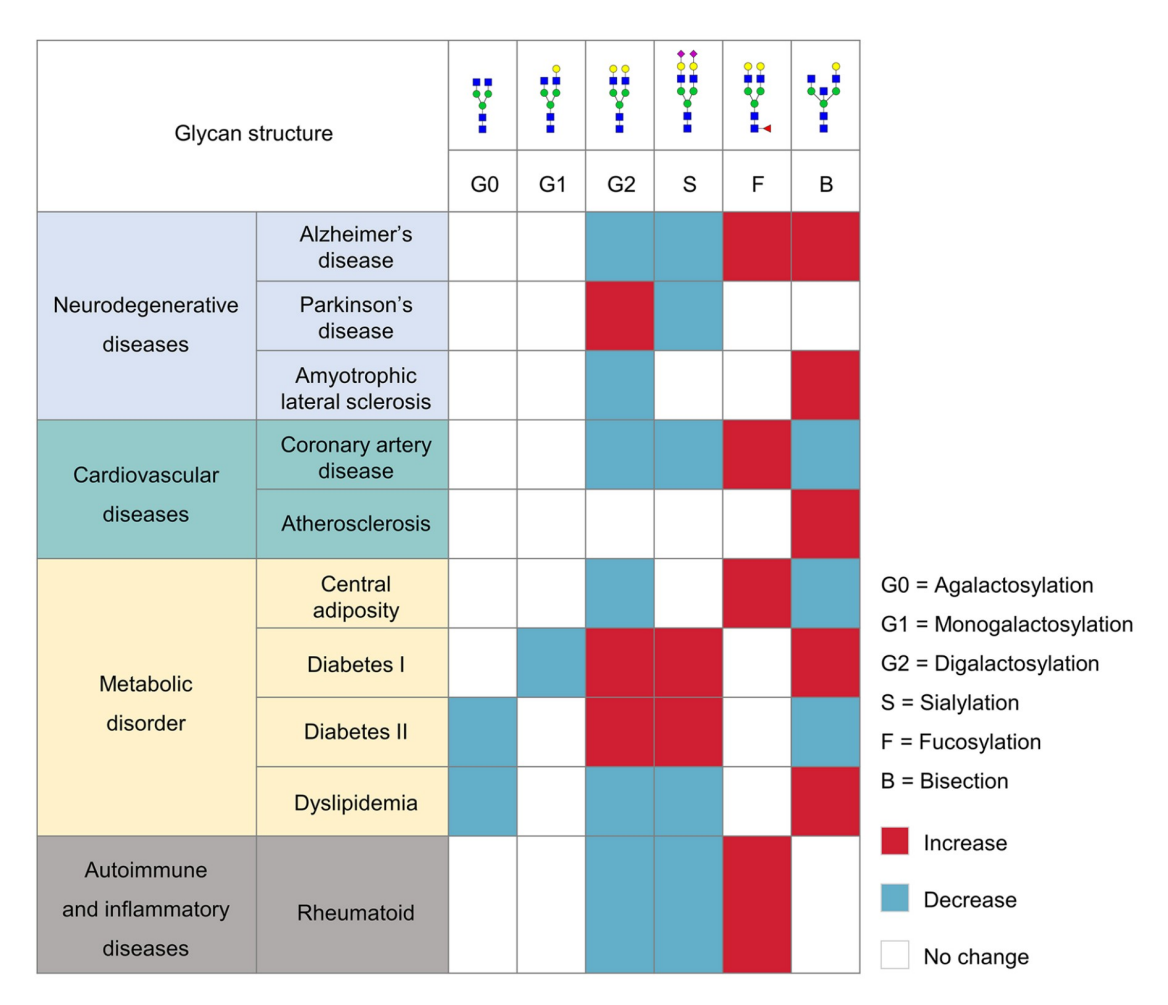
Figure 6 Changes in IgG glycosylation in aging-related diseases

Alterations in IgG glycosylation with aging have been implicated in the dysregulation of immune responses, impaired clearance of harmful molecules, and altered interactions with immune cells, thereby contributing to the pathogenesis of various age-related diseases ( Figure 6). Summarizing these IgG glycosylation alterations associated with different age-related diseases could enhance our comprehensive understanding of the functions of IgG glycosylation in various physiological and pathological conditions.

### Neurodegenerative diseases

Alterations in IgG glycosylation patterns are present in various neurodegenerative diseases, including Alzheimer’s disease (AD) [87], Parkinson’s disease (PD) [88], and amyotrophic lateral sclerosis (ALS) [89]. Specifically, changes in the composition and structure of IgG glycan molecules have been observed in the serum and cerebrospinal fluid of affected individuals.

In Alzheimer’s disease, aberrant IgG glycosylation has been associated with the aggregation and deposition of amyloid-beta plaques, a hallmark pathology of the disease. Research has shown that AD patients have an altered glycan profile, with an increase in fucosylated glycoforms and a decrease in galactosylated and sialylated glycoforms [87]. A case–control study revealed that decreased sialylation and core fucosylation and increased bisecting N-acetylglucosamine (GlcNAc) *N*-glycan structures were significantly different between patients with dementia and those with normal cognitive functioning [90]. These glycosylation changes may affect the clearance mechanisms responsible for removing amyloid-beta from the brain, thereby exacerbating its accumulation and contributing to neuronal dysfunction and cognitive decline. In addition, to determine whether serum glycopeptide analysis has the potential to identify a new diagnosis and prognosis of AD, researchers found that the fucosylation status of IgG was able to distinguish AD patients from healthy controls. Specifically, an increased abundance of non-fucosylated IgG1 and IgG2 was observed in AD patients compared with controls [91].

In Parkinson’s disease, similar alterations in IgG glycosylation have been linked to the misfolding and aggregation of alpha-synuclein protein, leading to the formation of Lewy bodies, which are characteristic pathological features of the disease. A reduced level of sialylation, particularly monosialylation, was observed in PD patients, with reduced relative abundances of biantennary digalactosylated monosialylated glycans (GP17), biantennary digalactosylated disialylated glycans (GP21), biantennary galactosylated sialylated glycans (FGS/(FG+FGS)), biantennary monogalactosylated monosialylated glycans (FG1S1/(FG1+ FG1S1)), and biantennary digalactosylated monosialylated glycans (FG2S1/(FG2+FG2S1+FG2S2)). These effects lead to reduced inhibition of Fcγ-RIIIa binding and elevated antibody-dependent cell cytotoxicity (ADCC) activation in PD [88]. Furthermore, in individuals with ALS, changes in IgG glycosylation have been associated with the dysregulation of immune responses and the activation of inflammatory pathways within the central nervous system. Alterations in IgG glycosylation have also been reported in patients with ALS, with reduced galactosylation and increased bisecting GlcNAc and IgG glycosylation differing between cerebrospinal fluid and serum [ 92, 93]. These alterations may contribute to the progressive degeneration of motor neurons observed in ALS patients.

### Cardiovascular disease

IgG glycans can be used to predict acute cardiovascular events, the composition of which correlates with the risk of cardiovascular disease (CVD). Agalactosylated, monogalactosylated, and sialylated *N*-glycans with a bisecting GlcNAc have a positive association with CVD, whereas sialylated glycans without a bisecting GlcNAc have a negative association [94]. Birukov *et al*. [95] investigated the IgG *N*-glycome composition in 2175 individuals from the EPIC-Potsdam cohort, including 417 patients with myocardial infarction (MI) and stroke. They discovered a sex-dependent association between the presence of IgG glycans and the incidence of MI/stroke. In males, a weighted score derived from FA2BG2S1 and FA2G2S2 was linked to an increased risk of CVD. Conversely, in females, the FA2[3]G1 structure on IgG showed an inverse association with CVD risk [ 96, 95].

Coronary artery disease (CAD) is the most common cardiovascular disease, and previous studies have shown a significant association between CAD and the *N*-glycosylation of IgG. Specifically, a sex-stratified analysis of 316 patients with coronary atherosclerosis and 156 subjects with clean coronaries revealed differences in the IgG *N*-glycome composition. Interestingly, the most significant differences were observed in women, where the presence of sialylated *N*-glycan structures was negatively correlated with CAD [97]. Abnormal IgG glycosylation has been associated with endothelial dysfunction, oxidative stress, and thrombotic events in coronary artery disease. These glycosylation alterations may influence the pro-inflammatory and pro-thrombotic properties of IgG antibodies, contributing to the pathogenesis of CAD [97].

In atherosclerosis, changes in IgG glycosylation have been linked to the inflammatory processes underlying plaque formation and progression. A cross-sectional study revealed that increased levels of bisecting GlcNAc in the IgG *N*-glycome were positively correlated with the presence of atherosclerotic plaques in carotid and femoral arteries. Conversely, sialylated glycans lacking bisecting GlcNAc were negatively associated with each other [94]. Dysregulated glycosylation of IgG antibodies may modulate their interactions with immune cells and circulating lipoproteins, exacerbating vascular inflammation and promoting atherosclerotic lesion development. Wu *et al*. [98] conducted a cross-sectional study enrolling 1465 individuals aged between 40 and 70 years from the Busselton Health and Aging Study. They employed machine learning techniques such as recursive feature elimination and penalized regression algorithms to systematically screen for significant glycans and construct an IgG *N*-glycosylation cardiovascular age (GlyCage) index. Notably, fucosylated *N*-glycans with bisecting GlcNAc (GP6, FA2B) and bis-galactosylated *N*-glycans with bifurcated GlcNAc (GP13, A2BG2) were found to make the most substantial contributions to the index.

### Metabolic disorders

The role of IgG glycosylation in metabolic diseases is increasingly recognized. Dysregulation of IgG glycosylation has been implicated in various metabolic disorders, including obesity, diabetes, and dyslipidemia [ 99‒ 101]. A high body mass index (BMI) is commonly used as a criterion for obesity, but when using the IgG glycome as a biomarker, the waist-to-hip ratio should be used instead of BMI, as it has been shown that individuals with a normal BMI who have central adiposity (as measured by the waist-to-hip ratio) have lower levels of galactosylation and bisecting GlcNAc and higher fucosylation [ 99, 102]. Importantly, hyposialylated IgG can activate the endothelial IgG receptor FcγRIIB to promote obesity-induced insulin resistance [100]. The common types of diabetes are type 1 diabetes mellitus (T1DM) and type 2 diabetes mellitus (T2DM). Studies have shown that the onset of type 1 diabetes is associated with an increase in the proportion of plasma and IgG high-mannose and bisecting GlcNAc structures, a decrease in monogalactosylation, and an increase in IgG disialylation [103]. Furthermore, Liu *et al*. [104] observed a decrease in bisecting N-acetylglucosamine of IgG2 and agalactosylation of IgG4 and an increase in sialylation of IgG4 and digalactosylation of IgG2 in T2DM patients. In addition, Plomp *et al*. [105] reported a positive association between IgG1 fucosylation and insulin levels and between IgG4 galactosylation and sialylation and insulin, as well as a positive association between IgG4 bisection and glucose. Age-related changes in IgG glycosylation in T2DM patients have been highlighted in several studies, and age-related increases in α2,3-linked and α2,6-linked sialylation on fucosylated glycans have been observed, suggesting a potential link between aging and chronic inflammation and disease exacerbation [106].

Dyslipidemia is another common metabolic disease and a key contributor to atherosclerosis. In dyslipidemia, inflammation is driven not only by abnormal glycosylation of lipoproteins but also by modifications of IgG glycans. Reductions in galactose and sialic acid residues, together with the introduction of bisecting GlcNAc on IgG glycans, may contribute to the persistent inflammation observed during the development and progression of dyslipidemia [ 101, 107]. Understanding the alterations in IgG glycans, such as changes in galactose and sialic acid residues and the presence of bisecting GlcNAc, provides insights into the underlying mechanisms of inflammation in conditions such as dyslipidemia.

### Autoimmune and inflammatory diseases

The activity and interaction of IgG, a critical component of humoral immunity, with other immune cells and molecules can be largely influenced by glycosylation. Rheumatoid arthritis (RA) was the first disease reported to be associated with altered IgG glycosylation [108]. A decrease in galactosylated and sialylated IgG glycans was found in patients with RA [ 109‒ 111]. Other researchers have used lectin microarrays to detect higher levels of fucosylation in the plasma of RA patients [112]. These glycosylation changes may affect the functional properties of IgG, such as antibody-dependent cell-mediated cytotoxicity (ADCC) and inflammatory regulation. Specifically, reduced galactosylation may reduce the anti-inflammatory function of IgG, whereas increased sialylation may enhance its anti-inflammatory effects [113]. In addition, the ratio of serum G0/G1 (non-galactosylated to monogalactosylated) can serve as a diagnostic marker to distinguish RA patients from healthy individuals as early as 3.5 years prior to the onset of the disease [114].

In a collagen-induced arthritis (CIA) model, genetic blockade of sialylation in activated B cells tends to increase joint inflammation. Conversely, artificial glycosylation of anti-type II collagen antibodies, including anti-citrullinated peptide antibodies (ACPAs), not only attenuates arthritis-inducing activity but also inhibits the development of CIA in antibody-pretreated mice. Notably, glycosylation of other IgGs does not prevent CIA [115]. In another study, TH17 cells regulated the expression of β-galactoside α2,6-sialyltransferase 1 in newly differentiated antibody-producing cells by directing B cells in an IL-22- and IL-21-dependent manner. This regulation determines the glycosylation profile and activity of IgG produced by subsequent plasma cells. Asymptomatic patients with RA-specific autoantibodies exhibit similar changes in the activity and glycosylation of autoreactive IgG antibodies before they enter the inflammatory phase of RA [116]. Additionally, certain autoimmune and inflammatory diseases, such as multiple sclerosis (MS) [117] and systemic lupus erythematosus (SLE) [118], exhibit significantly different IgG glycomic profiles. Although these conditions are influenced by age, they are primarily categorized as autoimmune diseases rather than aging-related diseases and thus will not be discussed in detail here.

Overall, the above studies on aging-related diseases suggest that IgG glycosylation may play a significant role in disease pathogenesis by influencing protein aggregation, immune responses, and chronic inflammation ( Table 1). Further investigation into the underlying mechanisms of IgG glycosylation dysregulation may provide insights into potential therapeutic targets for the treatment of these devastating conditions.

**Table:** **Table 1** Altered IgG glycosylation in age-related diseases

Diseases	Samples	Increased glycoforms	Decreased glycoforms	Year	Ref
AD	Plasma	Fucosylation	Galactosylation and sialylation	2014	[87]
Plasma	Bisecting GlcNAc	Sialylation and core fucosylation	2021	[90]
Plasma	Non‐fucosylated IgG1 and IgG2		2022	[91]
PD	Plasma	Galactosylation	Sialylation	2017	[88]
ALS	Plasma and cerebrospinal fluid	Bisecting GlcNAc	Galactosylation	2015	[93]
Cerebrospinal fluid		Galactosylation	2019	[92]
Cardiovascular disease	Plasma	Agalactosylated, monogalactosylated, and sialylated glycans with bisecting GlcNAc	Sialylated glycans without bisecting GlcNAc	2021	[119]
Plasma	Galactosylated and sialylated fucosylated glycoforms without bisecting GlcNAc		2022	[ 95, 96]
Atherosclerosis	Plasma	Bisecting GlcNAc		2018	[94]
Coronary artery disease	Plasma		Sialylation	2023	[97]
Central adiposity	Plasma	Fucosylation	Galactosylation and bisecting GlcNAc	2019	[ 99, 102]
Type 1 diabetes mellitus	Plasma	High-mannose, bisecting GlcNAc and disialylation	Monogalactosylation	2022	[103]
Plasma	Galactosylation and sialylation	Simple biantennary *N*-glycans	2018	[120]
Type 2 diabetes mellitus	Plasma	Sialylation of IgG4 and digalactosylation of IgG2	Agalactosylation of IgG4 and bisecting N-GlcNAc of IgG2	2019	[104]
Plasma	IgG4 bisection		2017	[105]
Dyslipidemia	Plasma	Bisecting GlcNAc	Galactosylation and sialylation	2018	[ 101, 107]
Rheumatoid arthritis	Plasma	Fucosylation	Galactosylation and sialylation	2023	[ 111, 112]

## Conclusions

The altered glycosylation patterns observed on IgG in aging-related diseases underscore the intricate interplay between immune function, inflammation, and aging processes. The distinct changes in IgG glycosylation profiles, including reductions in galactosylation and sialylation, alongside increases in agalactosylation and bisecting GlcNAc, serve as potential biomarkers for assessing aging-related pathologies. These glycan modifications reflect underlying physiological changes associated with aging, inflammation, and immune dysregulation, highlighting the importance of IgG glycosylation as a promising avenue for understanding and diagnosing aging-related diseases.

Further research is warranted to elucidate the mechanisms underlying alterations in IgG glycosylation in aging-related diseases and their functional implications. This includes investigating the causal relationships between specific glycan structures and disease pathogenesis, as well as exploring the potential of IgG glycan-based interventions for disease prevention and treatment. IgG glycosylation is strongly influenced by genetic and environmental factors, including age, gender, ethnicity and geographic distributions. It is therefore necessary to integrate IgG glycan biomarkers with other types of biomarkers discovered by genomics, proteomics, and metabolomics to comprehensively assess age-related phenotypes. This holistic approach could provide deeper insights into the complex interplay between molecular pathways underlying aging processes and open up avenues for the development of innovative interventions aimed at promoting healthy aging and extending lifespan.

In summary, the study of altered IgG glycosylation in aging-related diseases as well as during natural physiological aging holds significant promise for advancing our understanding of glycosylation functions and disease mechanisms, improving diagnostic strategies, and ultimately enhancing therapeutic interventions to promote healthy aging and mitigate age-related pathologies.
